# Metabolomics Unveiled the Accumulation Characteristics of Taste Compounds During the Development and Maturation of Litchi Fruit

**DOI:** 10.3390/foods14010144

**Published:** 2025-01-06

**Authors:** Nonghui Jiang, Wei Liu, Zhidan Xiao, Xu Xiang, Yun Zhong

**Affiliations:** Key Laboratory of South Subtropical Fruit Biology and Genetic Resource Utilization, Ministry of Agriculture and Rural Affairs, Guangdong Provincial Key Laboratory of Science and Technology Research on Fruit Tree, Institute of Fruit Tree Research, Guangdong Academy of Agricultural Sciences, Guangzhou 510640, China; liuwei1987ahnu@126.com (W.L.); xiaozhidan@gdaas.cn (Z.X.); xiangxu@gdaas.cn (X.X.); zhongyun@gdaas.cn (Y.Z.)

**Keywords:** litchi, metabolomics, taste compounds, development, mature

## Abstract

Litchi is one of the ancient fruits that originated in China, renowned for its high nutrition and rich flavor, and Xianjinfeng (XJF) stands as one of the most notable varieties in terms of its flavor. Investigating the metabolic changes in taste compounds during fruit development offers deeper insights into the formation patterns of fruit quality. In this study, we conducted extensive metabonomic research on the accumulation patterns of taste compounds (carbohydrates, organic acids, and amino acids) across three developmental stages of XJF litchi. A total of 238 taste metabolites were detected. Cluster analysis and PCA revealed significant changes in metabolite composition and content across different stages, closely correlating with the developmental phase. The abundance of total taste metabolites in stage S1 was notably lower than stages S2 and S3. The total abundance of sugar continued to rise, yet monosaccharides and disaccharides exhibited distinct behaviors, highlighting the characteristic accumulation of reducing sugars. Most organic acids demonstrated a notable downward trend, whereas the abundance of most essential and flavor-contributing amino acids showed an upward trend. The number of DAMs across the three stages followed the trend of S1 vs. S3 > S1 vs. S2 > S2 vs. S3. KEGG functional annotation and enrichment revealed that amino acid biosynthesis, D-amino acid metabolism, 2-oxocarboxylic acid metabolism, glyoxylate and dicarboxylate metabolism, the pentose phosphate pathway, the tricarboxylic acid cycle, and carbon metabolism were the most significantly enriched primary metabolic pathways. More differential metabolites and metabolic pathways indicated that the critical stage from the green fruit stage to the color transition stage laid a solid foundation for litchi flavor. This experiment will offer valuable references for cultivation, breeding, processing, and consumption.

## 1. Introduction

Litchi (*Litchi chinensis* Sonn.) is a typical subtropical evergreen fruit tree that has been cultivated in China for over 2000 years. In 2023, the nationwide area of litchi in production reached 526.75 thousand hectares, ranking first in the world in terms of both cultivation area and yield [1]. Litchi boasts a unique taste [2], and among the numerous excellent varieties, ‘Xianjinfeng’ (XJF), a new variety approved by the Guangdong Provincial Variety Approval Committee of China in 2011, stands out as a favorite among consumers. This variety is characterized by its bright red fruit color, large size, sweet taste, high yield, storage stability, and resistance to cracking [3]. It has been promoted across the country over an area of 10 thousand hectares, making it the most widely promoted and beneficial new litchi variety in China in recent years. As export trade increases, it is gradually gaining recognition among consumers worldwide. As the public pursues a healthy lifestyle, there has been an increased focus on the nutrition and taste of fruits. Researchers have explored and summarized the patterns of nutritional quality changes during the development of various fruits [4,5,6]. The flavor of litchi is determined by both taste and odor. Sugar, acid, and amino acids serve not only as essential nutrients but also as primary taste compounds, providing a crucial basis for consumer choice [7]. However, the ripening process of litchi is accompanied by significant changes in flavor and color, which impact fruit quality. Therefore, studying the taste compounds during the development and ripening of litchi helps us better understand the formation patterns of fruit quality. Current studies have revealed that the development of litchi fruit, from pollination to maturity, takes approximately 60–100 d and follows a single S-shaped growth curve [8]. Additionally, they have identified some metabolite synthesis, degradation, and other functional phytochemicals related to fruit quality [9,10]. Currently, metabolomics technology, utilizing ultra-performance liquid chromatography–mass spectrometry (UPLC-MS/MS), has been employed to decode the metabolic changes during fruit development in various plants, owing to its advantages such as high throughput, panoramic analysis, and technical flexibility [11,12]. A series of studies have been conducted on the cultivation methods of XJF litchi in recent years [13,14]. Some quality indicators of XJF litchi have been evaluated using conventional methods [15]. However, there is still a lack of in-depth research on the formation of taste quality in this distinctive late-ripening litchi variety, utilizing extensive targeted metabolomics technology.

In this study, we utilized the extensive targeted metabolomics approach based on UPLC-MS/MS to assess the wide range of metabolite changes during the development and maturation of XJF litchi fruit. We conducted a comparative analysis of the types and abundances of three kinds of metabolites (referred to as taste metabolites), namely carbohydrates, organic acids, and amino acids. Additionally, we compared the differences in metabolites and metabolic pathways across various developmental stages, aiming to gain a deeper understanding of the dynamic changes in taste compounds during the development and maturation of litchi fruit. This provides a crucial foundation for litchi cultivation, breeding, processing, and consumption.

## 2. Materials and Methods

### 2.1. Materials and Treatment Methods

The samples were collected from the National Litchi Germplasm Resources Garden located in Tianhe District, Guangzhou, Guangdong Province, China. This garden is primarily dedicated to the collection, preservation, and utilization research of litchi varieties. The variety selected was XJF. Samples were collected at 55 days, 63 days, 66 days, 70 days, 74 days, 80 days, and 85 days after the female flowers bloomed. Fruits of uniform shape, size, and color were packaged in airtight bags and promptly transported to the laboratory (within 0.5 h). The pulp samples were then collected and frozen in liquid nitrogen for fruit quality analysis. Metabolomic tests were conducted at three developmental stages of 55 days, 70 days, and 85 days mentioned above, denoted as S1 (green fruit stage, with the aril just fully wrapping around the fruit core), S2 (color transition stage, with yellow pericarp about to turn red), and S3 (mature stage, 8–9 maturity with bright red pericarp). Each of the samples included three biological replicates.

### 2.2. Sample Preparation and Analysis

Metabolomic samples were prepared according to Chen’s method [16]. The litchi delivered to the laboratory was removed from the pericarp and core, and only the pulp was selected. The freeze-dried (−20 °C) pulp tissue was ground (30 Hz, 1.5 min) to powder using a Heto Lyolab 3000 vacuum freeze-drier (Heto-Holten, Allerød, Denmark) and a grinder (Retsch, Haan, Germany). A total of 100 mg of powder was accurately weighed, extracted in 1.0 mL of 70% methanol aqueous solution, and refrigerated overnight at 4 °C, during which it was rotated three times. After centrifugation (10,000× *g*, 10 min) with a high-speed freezing centrifuge (Eppendorf, Hamburg, Germany), the supernatant was collected and filtered through a 0.22 μm microporous membrane, and then analyzed by UPLC-MS/MS.

UPLC-MS/MS was performed using a Shim-pack UFLC CBM30A system (Shimadzu, Kyoto, Japan) coupled with a 4500 QTRAP tandem mass spectrometer (Applied Biosystems, Foster City, CA, USA). HPLC conditions, mobile phase, gradient program, and mass spectrometry conditions refer to Chen’s method [16]. Electrospray ionization (ESI) was employed for MS analysis, with a gas curtain pressure of 25 psi and collision-induced ionization set to high. In the triple quadrupole, each ion pair was scanned based on optimized de-clustering potentials and collision energy.

The content of total soluble solids (TSS) is measured using a pocket refractometer (Atago, Kyoto, Japan), the content of titrable acid (TA) and vitamin C (Vc) is measured in accordance with the Chinese national standard [17,18]. 

### 2.3. Qualitative and Quantitative Data Analysis of Metabolites

Based on the MetWare Database (MWDB), substances were qualitatively identified based on secondary spectral information. Metabolite quantification was accomplished through multiple reaction monitoring (MRM) analysis using triple quadrupole mass spectrometry. The mass spectrum data were processed using Analyst 1.6.3 software. We obtained the total ion current (TIC) diagram and the MRM metabolite detection multi-peak diagram for quality control (QC) samples. Multidimensional statistical analysis (Variable Importance in Projection, VIP), one-dimensional statistical analysis (*p*-value), and fold change (FC) were employed to predict the stability and reliability of the model. Metabolites with VIP > 1, log2FC ≥ 2, or log2FC ≤ 0.5 were identified as differential accumulated metabolites (DAMs).

### 2.4. Statistical Analysis

Principal component analysis (PCA), hierarchical cluster analysis (HCA), and orthogonal partial least squares discriminant analysis (OPLS-DA) were utilized to predict the stability and reliability of the model. Unsupervised PCA was performed by the statistics function prcomp within R (www.r-project.org, accessed on 10 May 2024). The data were unit variance-scaled before unsupervised PCA. The HCA results of samples and metabolites were presented as heatmaps with dendrograms, while Pearson correlation coefficients (PCC) between samples were calculated by the cor function in R and were presented as only heatmaps. Both HCA and PCC were carried out with ComplexHeatmap package in R software.

Additionally, Graphpad Prism 8.0 and IBM SPSS 25 software were employed for statistical analysis and chart creation. Duncan’s new multiple range test was used for comparisons between sample groups, where *p* < 0.05 and *p* < 0.01 denoted significant and extremely significant differences, respectively.

## 3. Results

### 3.1. Some Phenotypic and Quality Characteristics of Fruit Development

**Figure 1:**
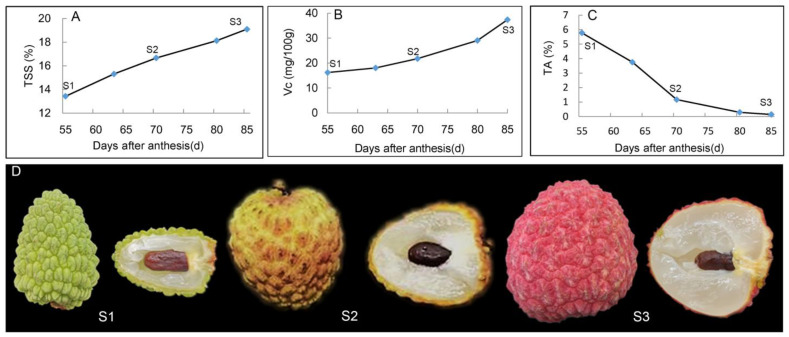
Changes in appearance and quality of XJF litchi at different developmental stages: (**A**) changes of the content of total soluble solids, (**B**) changes of the content of vitamin C, (**C**) changes of the content of titratable acid, and (**D**) appearance and cross-section.

As the XJF litchi fruit develops, the trends in the contents of TSS, Vc, and TA vary. Both the TSS and Vc contents exhibited an upward trend (Figure 1A,B). The TSS content rose by 3.24% prior to color transition (between 55 and 70 days after flowering), but only increased by 2.44% during the later stage of color transition (from 70 to 85 days after flowering). Conversely, Vc content increased more rapidly in the later stage of color transition, rising by 5.58 mg/100g before color transition and by 15.59 mg/100g during the later stage of color transition. The TA content followed a downward trend, plummeting from 5.77% to 1.17%, and further dropping to 0.14% after color change, signifying an even greater decrease (Figure 1C). Additionally, the peel color underwent a transformation from green during the green fruit stage to yellow during the color transition stage, and finally to bright red at maturity (Figure 1D). The peel color serves as an intuitive indicator of fruit ripeness.

### 3.2. Overview of Metabonomic Analysis

**Figure 2:**
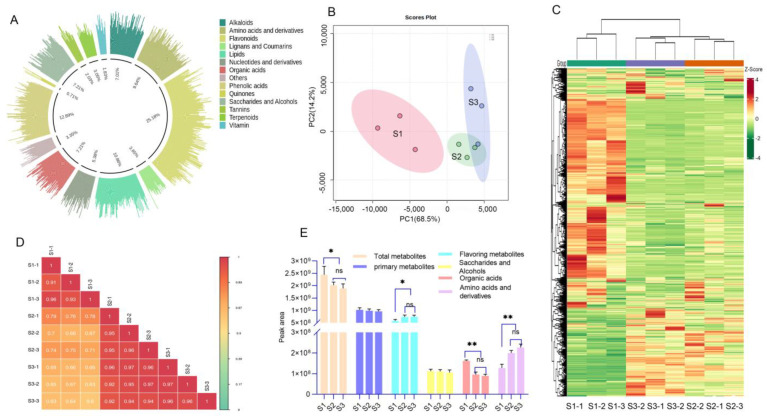
Overview of the litchi metabolome at different developmental stages: (**A**) classification diagram, (**B**) PCA diagram, (**C**) cluster heatmap, red represents high content, green represents low content, (**D**) correlation diagram between samples, different colors represent varying magnitudes of Pearson correlation coefficients, a redder color indicates a stronger positive correlation, a greener color signifies a weaker correlation, and (**E**) summary and comparison of peak areas for various metabolites, * *p* < 0.05, ** *p* < 0.01, ns: no significance.

Extensive targeted metabonomic studies identified 985 metabolites in the pulp, including 238 taste metabolites, with 71 saccharides and alcohol, 72 organic acids, and 95 amino acids (Table S1, Figure 2A). To explore the correlation between changes in metabolite expression patterns and fruit development stages, we conducted a PCA on metabolites from samples across three developmental stages (Figure 2B). The results indicated that samples from the same developmental stage were clustered together. The sum of the principal components of PC1 and PC2 metabolites accounted for 82.7% of the total variance, with a clear separation trend observed between S1 and S2, as well as S1 and S3. We generated a cluster heatmap, illustrating the results of HCA of metabolites across the three developmental stages and categorizing metabolites with similar characteristics (Figure 2C). The findings revealed distinct grouping patterns among different developmental stages, indicating significant changes in metabolite composition and content, with high reliability and reproducibility of the data. The correlation analysis chart (Figure 2D), derived from the Pearson correlation coefficient, illustrates the correlation among different developmental stages: S1 vs. S3 < S1 vs. S2 < S2 vs. S3.

By summarizing the peak areas of metabolites within the same category, we calculated the total peak area of taste metabolites, primary metabolites, and total metabolites to assess their ion abundances (Figure 2E). Notably, the total metabolite abundance in S1 was significantly higher compared to S2 and S3 (*p* < 0.05). The total taste metabolite abundance of S1 was significantly lower than that of S2 and S3 (*p* < 0.05). Comparing the three taste metabolites, it was found that there were no significant differences in the total abundance of saccharides and alcohols across the three developmental stages (*p* > 0.05). Meanwhile, the total abundance of organic acids in the S1 stage was significantly higher than that in the S2 and S3 stages (*p* < 0.01). Additionally, the total abundance of amino acids showed an upward trend across all stages, with the S3 and S2 stages being significantly higher than the S1 stage (*p* < 0.01).

### 3.3. Variation Characteristics of Nonvolatile Taste Metabolites at Different Development Stages

#### 3.3.1. Accumulation Characteristics of Saccharides and Alcohols

**Figure 3:**
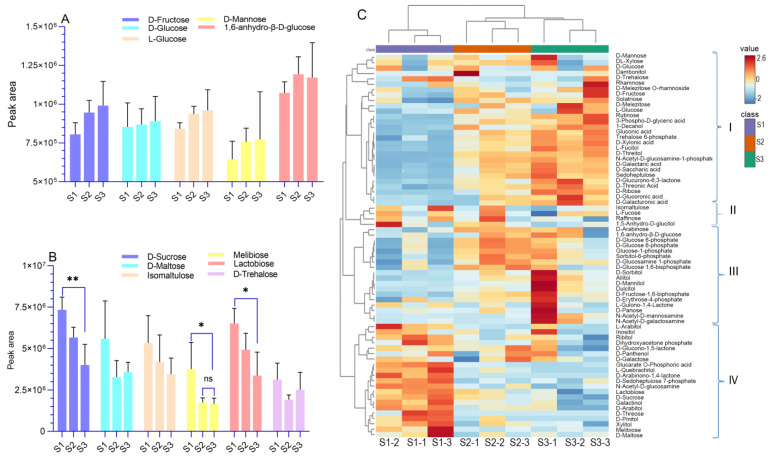
Comparison of peak areas of saccharides and alcohol metabolites in litchi at different developmental stages: (**A**) monosaccharides, (**B**) disaccharides, and (**C**) cluster heatmap of saccharides and alcohol metabolites. * *p* < 0.05, ** *p* < 0.01, ns: no significance.

XJF litchi is rich in diverse sugars, including 13 monosaccharides, 7 disaccharides, 3 trisaccharides, 11 saccharide alcohols, 14 phosphate sugars, and 7 sugar acids. These compounds exist in various forms, such as free states, oligosaccharides, and derivatives resulting from alcoholization, phosphorylation, amination, acidification, esterification, and aldehyde reactions. The cluster heatmap of saccharides and alcohols reveals the trends of each substance, which are clustered into four categories based on the abundance variations of each component (Figure 3C). Most monosaccharide compounds, including mannose, DL-Xylose, L-glucose, D-fructose, D-xyloic acid, L-fucitol, D-threitol, D-glucuronic acid, D-galacturonic acid, and Sedoheptulose fall into Category I. Their abundance shows an upward trend across three developmental stages, although this trend is not significant (*p* > 0.05, Figure 3A). Most disaccharides, such as D-sucrose, D-maltose, melibiose, and lactobiose are grouped in Category IV. Here, S1 consistently exceeds S2 and S3, showing a downward trend (Figure 3B,C). Most phosphate-sugar compounds (including glucose-1-phosphate, D-glucosamine-1-phosphate, sorbitol-6-phosphate, D-glucose-1,6-diphosphate, D-glucose-6-phosphate, and D-fructose-6-phosphate) are categorized in Category III. These compounds exhibit high abundance in S2 and often serve as intermediate products in glucose metabolism. The results indicated that monosaccharides in the fruit accumulated continuously from the green fruit stage to the maturity stage, reaching a peak at maturity. The accumulation of disaccharides was already at a high peak during the green fruit stage, while intermediate phosphate-sugar compounds typically accumulated to a certain peak during the color transition stage.

#### 3.3.2. Accumulation Characteristics of Organic Acid Metabolites

**Figure 4:**
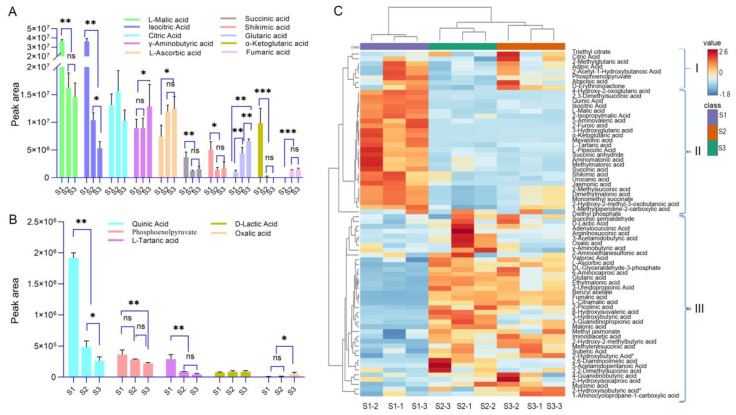
Comparison of peak areas of organic acid metabolites in litchi at different developmental stages: (**A**) major organic acids with high abundance, (**B**) other organic acids, and (**C**) cluster heatmap of organic acids. * *p* < 0.05, ** *p* < 0.01, *** *p* < 0.001, ns: no significance.

We discovered that the types of organic acids in XJF litchi are complex and diverse, with both free organic acids and organic acid derivatives coexisting. The main organic acids with high abundance include L-malic acid, isocitric acid, citric acid, γ-aminobutyric acid, ascorbic acid, glutaric acid, α-ketoglutarate, shikimic acid, succinic acid, and fumaric acid (Figure 4A). Each organic acid was clustered into four major categories based on its variation in abundance (Figure 4C). In Category II of the heatmap, the abundance values of L-malic acid, succinic acid, L-tartaric acid, α-ketoglutarate, shikimic acid, quinic acid, isocitric acid, and jasmonic acid were significantly higher in S1 compared to S2 and S3 (*p* < 0.05, Figure 4C). This indicates a significant downward trend in the accumulation of these organic acids as the fruit develops, particularly from the green fruit stage to the color transition stage. Notably, L-malic acid, isocitric acid, α-ketoglutarate, phosphoenolpyruvic acid, and quinic acid exhibited a downward trend across all stages. Conversely, in Category IV, organic acids such as ascorbic acid, γ-aminobutyric acid, fumaric acid, lactic acid, and oxalic acid accumulated to their peak at stage S3 (Figure 4A,B). These findings suggest that XJF litchi is rich in L-malic acid, succinic acid, α-ketoglutarate, fumaric acid, γ-aminobutyric acid, ascorbic acid, citric acid, and its isomers.

#### 3.3.3. Accumulation Characteristics of Amino Acid Metabolites

**Figure 5:**
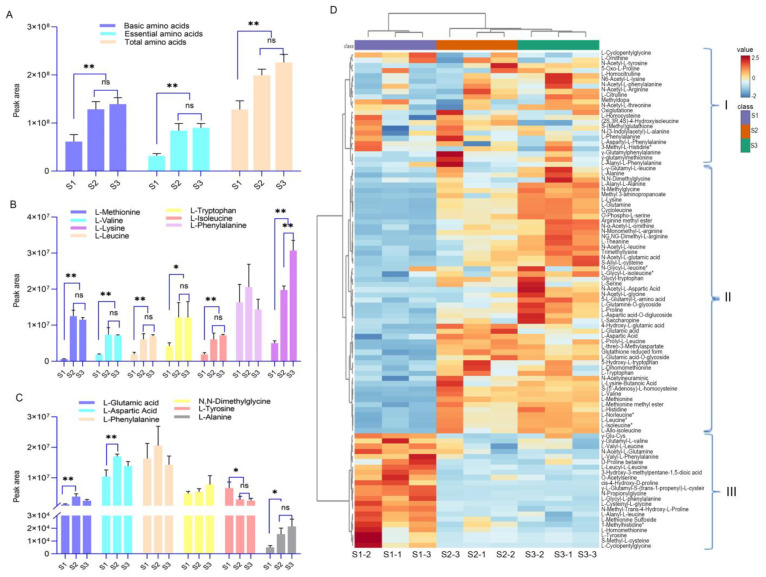
Comparison of peak areas of amino acid metabolites in litchi at different developmental stages: (**A**) overview of amino acids, (**B**) essential amino acids, (**C**) taste-related amino acids, and (**D**) cluster heatmap of amino acid metabolites. * *p* < 0.05, ** *p* < 0.01, ns: no significance.

The type and content of amino acids in food serve as crucial indicators for assessing its nutritional quality and sensory taste. Glutamic acid, aspartic acid, phenylalanine, alanine, tyrosine, glycine, and their sodium salts are capable of imparting a distinctive savory or sweet taste to food, collectively known as flavor amino acids [19]. This study discovered that XJF litchi comprises 95 amino acid metabolites, encompassing 19 basic amino acids (excluding asparagine) and 7 essential amino acids. During the three developmental stages of XJF litchi, the abundance of basic, essential, and total amino acids exhibited an upward trend, with S2 and S3 significantly surpassing S1 (*p* < 0.01) (Figure 5A). Based on the abundance of each component, the cluster heatmap clustered them into three major categories (Figure 5D). The proportions of basic amino acids in the three developmental stages, accounting for the total amino acid content, are 33.54%, 70.87%, and 77.67%, respectively. Among the seven detected essential amino acids, with the exception of phenylalanine, which showed no significant variation, most others clustered in Category II of the heatmap, the abundances of S3 and S2 were significantly higher than S1 (*p* < 0.01, Figure 5B). The proportions of essential amino acids in the overall amino acid content were 17.16%, 45.69%, and 48.73% across the three stages, respectively.

In this experiment, five free taste-related amino acids (glutamic acid, aspartic acid, phenylalanine, alanine, and tyrosine) were identified, among which L-glutamic acid, L-aspartic acid, and glycine fell into Category II of the cluster heatmap, with S2 significantly higher than S1. Conversely, L-tyrosine was in Category III, with S1 notably higher than S2 and S3 (*p* < 0.05, Figure 5C). Alanine and the derivatives of glycine (N,N-Dimethylglycine) exhibited an upward trend across the three stages. Notably, this experiment also revealed a significant increase in the abundance of theanine, a sweet-tasting amino acid, with developmental progression (*p* < 0.05, Figure 5C). Theanine is known to alleviate anxiety, enhance mood, improve cognition, and promote sleep [20].

### 3.4. Analysis of Significant Differences

To investigate the variations in metabolites during different developmental stages of litchi fruit, the differential accumulated metabolites (DAMs) of XJF were analyzed. Samples from various developmental stages were divided into three groups: S1 vs. S2, S2 vs. S3, and S1 vs. S3, for OPLS-DA comparison. An OPLS-DA model was established (Figure S1). The R2Y values for the three comparison groups were all ≥0.999, R2X values were ≥0.517, and Q2 values were ≥0.765, indicating the effectiveness of the OPLS-DA model. Based on the OPLS-DA results, DAMs for each group were selected.

**Figure 6:**
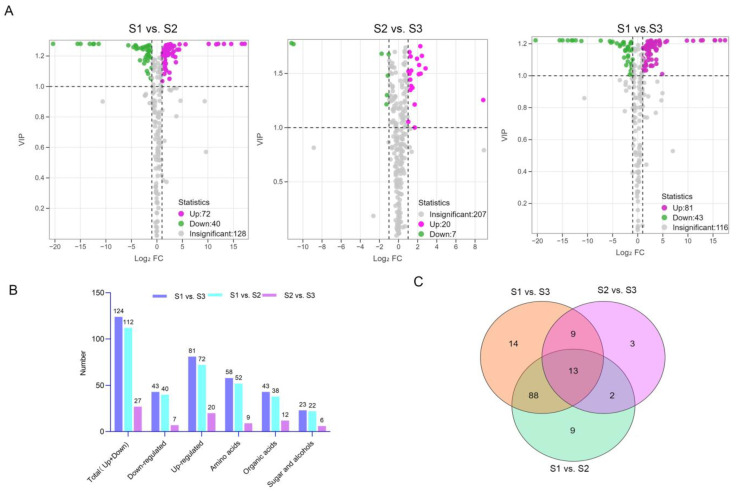
Comparison of DAMs of taste metabolites in XJF litchi at different developmental stages: (**A**) volcano plot, the green dot represents the down-regulated DAMs, the red dot represents the up-regulated DAMs, the gray dots represent detected but insignificant metabolites, (**B**) statistics of DAMs, and (**C**) Venn diagram, the numbers in the overlapping parts of the circles represent the number of common DAMs between the comparison groups, the numbers without overlapping parts represent the number of unique DAMs in the comparison groups.

The volcanic map of DAMs (Figure 6A) provides a visual representation of their overall distribution, revealing a quantity trend of S1 vs. S3 > S1 vs. S2 > S2 vs. S3 (Figure 6B). Specifically, S1 vs. S2 yielded a total of 112 DAMs of the three types of taste metabolites (Table S2), with 72 up-regulated and 40 down-regulated metabolites; 52 types of amino acids comprised the majority, followed by 36 types of organic acids, and then 22 types of saccharides and alcohols. Most free amino acids, basic amino acids such as L-methionine and L-lysine, and certain amino acid derivatives showed up-regulation, whereas only a few amino acid dimers and derivatives, such as L-cysteinyl-L-glycine, were down-regulated. Among the organic acids, 15 types including fumaric acid and glutaric acid exhibited up-regulation, whereas 20 types such as L-malic acid, L-tartaric acid, α-ketoglutarate, isocitric acid, quinic acid, succinic acid, shikimic acid showed down-regulation, with the number of down-regulated acids slightly exceeding the up-regulated ones. In the saccharide and alcohol categories, most monosaccharides and their derivatives, such as D-ribose, demonstrated up-regulation, while only a handful of metabolites, including melibiose and D-arabitol, were down-regulated. A total of 27 types of DAMs of taste metabolites were obtained in S2 vs. S3 (Table S3), with 20 up-regulated and 7 down-regulated metabolites; 12 types of organic acids were the most prevalent, followed by 9 types of amino acids, and then saccharides and alcohols (Figure 6B). Among the amino acids, only L-cysteinyl-L-glycine was down-regulated, while the rest (including L-theanine) were up-regulated. Among the organic acids, oxalic acid and the other eight types were up-regulated, while α-ketoglutarate was down-regulated. In saccharides and alcohols, D-ribose and D-mannitol were up-regulated, while D-arabitol and L-quebrachitol were down-regulated. In S1 vs. S3 (Table S4), a total of 124 DAMs of taste metabolites were identified (81 up-regulated and 43 down-regulated). It was similar to that in S1 vs. S2, with amino acids being the most prevalent (58 types), followed by organic acids (43 types), and then saccharides and alcohols (23 types). The variation in the number of DAMs indicates that more taste metabolites were involved in complex biochemical reactions from the green fruit stage to the color transition stage. The changes in litchi taste primarily occurred during this period, with the number of organic acids and amino acids significantly exceeding that of saccharides. 

The Wayne diagram (Figure 6C) reveals that there are 13 common DAMs across the three control groups, including 3 saccharides and alcohols (L-quebrachitol, D-ribose, D-mannitol), 5 organic acids (α-ketoglutaric acid, 3-hydroxyglutaric acid, 3-hydroxybutyric acid, β-hydroxyisovaleric acid, 2,6-diaminoheptanoic acid), and 5 amino acids (L-cysteinyl-L-glycine, L-alanyl-L-alanine, L-theanine, NG,NG-dimethyl-L-arginine, L-glutamine-O-glucoside). These common DAMs may serve as key metabolites regulating the sweet and sour taste of XJF litchi at various developmental stages.

### 3.5. KEGG Function Notes and Enrichment Analysis

**Figure 7:**
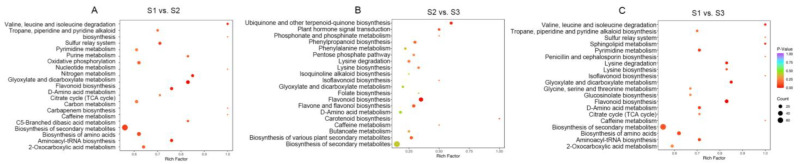
KEGG differential enrichment bubble chart: (**A**) S1 vs. S2, (**B**) S2 vs. S3, (**C**) S1 vs. S3,the abscissa represents the Rich Factor for each pathway, the ordinate shows the pathway name, the color of the points reflects the *p*-value, with a redder dot indicating a more significant enrichment, and the size of the points indicates the number of enriched DAMs.

Based on the DAMs results, a KEGG pathway enrichment analysis was conducted. The KEGG enrichment diagram (Figure 7) illustrates the 20 most significantly enriched pathways for each group. In groups S1 vs. S2, 155 DAMs were annotated by KEGG into 86 pathways, with the most significantly enriched metabolic pathways primarily involving secondary metabolite biosynthesis, amino acid biosynthesis, nucleotide metabolism, flavonoid biosynthesis, and carbon metabolism. In groups S2 vs. S3, 42 DAMs were annotated by KEGG into 50 pathways, with the most significantly enriched metabolic pathways mainly involving secondary metabolite biosynthesis, flavone biosynthesis, flavone and flavonol biosynthesis, D-amino acid metabolism, lysine degradation, and the pentose phosphate pathway. In groups S1 vs. S3, 159 DAMs were annotated by KEGG into 87 pathways, with the most significantly enriched metabolic pathways primarily involving secondary metabolite biosynthesis, amino acid biosynthesis, flavone biosynthesis, 2-oxocarboxylic acid metabolism, and D-amino acid metabolism. These most significantly enriched pathways represent the most active biological changes during each developmental stage of XJF litchi.

Table S6 presents the metabolic pathways related to saccharides, organic acids, amino acids, and the taste quality in XJF litchi. In groups S1 vs. S2, there are 17 pathways related to saccharides and organic acid metabolism. The most significantly enriched pathways primarily involve glycolysis/glyconeogenesis, galactose metabolism, fructose and mannose metabolism, pentose and glucuronic acid interconversion, the citric acid cycle, ascorbic acid, and aldehyde ester metabolism. Additionally, there are 18 pathways related to amino acid metabolism, with the most significantly enriched pathway involving amino acid biosynthesis, D-amino acid metabolism, cysteine and methionine metabolism, alanine, aspartate, and glutamate metabolism, glutathione metabolism, and arginine and proline metabolism. In groups S2 vs. S3, there were 14 pathways related to carbohydrate and organic acid metabolism, with the most significant enrichment pathways involving the mutual transformation of pentose and glucuronic acid, the pentose phosphate pathway, fructose and mannose metabolism, the citric acid cycle, pyruvate metabolism, and glyoxylate and dicarboxylate metabolism. Additionally, there were 14 pathways related to amino acid metabolism, with the most significant enrichment pathways involving amino acid biosynthesis, D-amino acid metabolism, glutathione metabolism, alanine, aspartate, and glutamate metabolism, lysine degradation, and arginine and proline metabolism. In groups S1 vs. S3, there were 19 pathways related to carbohydrate and organic acid metabolism, with the most significant enrichment pathways mainly involving carbon metabolism, the pentose phosphate pathway, glycosylphosphatidylinositol (GPI)-anchored biosynthesis, 2-oxocarboxylic acid metabolism, glyoxylate and dicarboxylate metabolism, and the citric acid cycle. There were also 18 pathways related to amino acid metabolism, with the most significant enrichment pathway involving D-amino acid metabolism, lysine biosynthesis, lysine degradation, valine, leucine, and isoleucine biosynthesis and degradation, and glycine, serine, and threonine metabolism. These most significantly enriched pathways represent the most active biological changes related to the sweet and sour taste of litchi.

The KEGG difference abundance score (DA score) captures the overall changes in all DAMs within a specific pathway. In the groups, the DA score is >0 for most amino acid and carbohydrate metabolism-related pathways, indicating an overall upward trend for these pathways during various developmental stages. Conversely, in most organic acid pathways, such as the citric acid cycle (S1 vs. S2, S2 vs. S3, and S1 vs. S3) and pyruvate metabolism (S1 vs. S2, S1 vs. S3), the DA scores are <0, suggesting a general downward trend for these pathways.

In the most significant enrichment pathway, the DAMs annotated in groups S1 vs. S2 were significantly more numerous than those in group S2 vs. S3, further proving that a greater number of DAMs were involved in metabolic changes during the transition from the green fruit stage to the color transition stage. Upon searching for DAMs associated with more than 10 metabolic pathways, compounds included L-glutamic acid, α-ketoglutarate, succinic acid, L-serine, L-glutamine, L-alanine, L-valine, fumaric acid, 3-phosphate-D-glyceric acid, and L-isoleucine, these compounds could be key substances involved in the biochemical reactions responsible for the sour and sweet flavor metabolism in XJF litchi.

## 4. Discussion

The changes occurring during each stage of fruit development, particularly those surrounding expansion, color transition, and maturation, are crucial. These stage-specific changes determine the ultimate commercial value of the fruit.

### 4.1. Sugars, Organic Acids, and Amino Acids Collectively Contribute to the Savory Taste of XJF Litchi

The soluble sugars in litchi pulp primarily consist of sucrose, glucose, and fructose [21]. Varieties of litchi exhibit distinct proportions of sugar components, manifesting various sugar accumulation patterns, including sucrose accumulation, intermediate accumulation, and reduced sugar accumulation [22]. In the XJF litchi variety, we observed a remarkable diversity in sugar types. During development, monosaccharides and disaccharides demonstrated distinct trends [23]. Most monosaccharides persistently accumulate from the green fruit stage to the fully ripe stage, peaking at maturity. However, disaccharides like D-sucrose, D-maltose, melibiose, and lactulose already accumulated significantly during the green fruit stage. Distinguishing it from melon [24], apple [25], and mulberry [26], the mid-to-late stages of maturity in XJF litchi are characterized by a rapid accumulation of reducing sugars accompanied by the degradation of disaccharides. Similar observations have been made in studies on tomatoes, ‘Feizixiao’, ‘Guiwei’, ‘Nuomici’, and ‘Groff’ litchis [21,23,27,28], indicating that XJF litchi possesses a unique characteristic of reducing sugar accumulation.

It is widely recognized that malic acid constitutes the majority of organic acids in litchi [22,29], yet there remains some controversy among scholars regarding organic acids other than malic acid in litchi [30,31,32]. We discovered that the abundance of L-malic acid, succinic acid, α-ketoglutaric acid, fumaric acid, γ-aminobutyric acid, ascorbic acid, and citric acid and its isomer is high in XJF litchi, whereas tartaric acid, oxalic acid, and lactic acid are present in lower abundance. As the fruit develops, the titratable acid content decreases, particularly from the green fruit stage to the color transition stage, with a declining rate approaching 80%, and remains at a very low level during the late maturation stage. The various organic acid components exhibited distinct trends; for instance, L-malic acid, succinic acid, L-tartaric acid, α-ketoglutaric acid, shikimic acid, quinic acid, and isocitric acid showed a significant downward trend, whereas ascorbic acid, γ-aminobutyric acid, fumaric acid, lactic acid, and oxalic acid exhibited the opposite trend. γ-aminobutyric acid (GABA), which is both an organic acid and a non-protein amino acid, serves as a neurotransmitter in the central nervous system and possesses benefits such as delaying aging and lowering blood pressure [33]; its rapid increase with fruit maturation facilitates the realization of these benefits in litchi. The decline in organic acid content during the late stage of fruit maturation can be attributed to various factors, including the rapid increase in fruit cell growth quality, the influx of a large amount of water, the decomposition of organic acids exceeding their synthesis, and their role as substrates in respiration and gluconeogenesis [34].

Free amino acids serve as precursors for the synthesis of crucial taste compounds in litchi fruit [35]. In our experiment, the total amino acid content in XJF litchi was notably low during the green fruit stage, but significantly increased as the fruit underwent color transition and ripening. Similar observations were made during the development of mango and mulberry [36,37]. The abundance of most essential amino acids during the color transition and ripening stages was significantly higher compared to the green fruit stage. The trend of taste-related amino acids in XJF litchi exhibited slight variations; notably, L-glutamic acid, L-aspartic acid, and aromatic phenylalanine reached their peak during the color transition period. Meanwhile, sweet alanine and the derivative of glycine exhibited an upward trend across all three stages. The accumulation of saccharides and taste-related amino acids, coupled with the decrease in organic acids, collectively contribute to the sweet and sour taste of XJF litchi.

### 4.2. Key Metabolites and Metabolic Pathways During Different Developmental Stages of Litchi

Our study revealed that carbon metabolism, the pentose phosphate pathway, the citric acid cycle, glyoxylate and dicarboxylate metabolism, biosynthesis of amino acids, and the metabolism of alanine, aspartic acid, and glutamic acid are the most prominent metabolic pathways that represent a series of highly active metabolic changes related to the taste quality. Additionally, fructose and mannose metabolism were enriched across all stages, with the most significant enrichment observed from the color transition stage to the maturity stage.

The pentose phosphate pathway serves as a means of oxidative decomposition for glucose. In vivo, apart from providing energy, the pentose phosphate pathway primarily supplies various raw materials for anabolism [38]. During the transition from the green fruit stage to the color transition stage, intermediate products of the pentose phosphate pathway such as D-fructose-1,6-diphosphate, 3-phosphate-D-glyceric acid, D-ribose, and gluconic acid were significantly up-regulated. Similarly, from the color transition stage to the maturity stage, 2-deoxyribose-5′-phosphate and D-ribose were up-regulated. 3-phosphate-D-glyceric acid is a crucial intermediate product in glycolysis and gluconeogenesis, playing a significant role [39]. The decrease in sucrose was accompanied by an increase in fructose, glucose, and other reducing sugars, suggesting a conversion of some sucrose into reducing sugars.

The organic acids constitute a crucial component of the citric acid cycle which plays a significant role in energy metabolism, gluconeogenesis, lipogenesis, and amino acid synthesis [27]. In this experiment, most intermediates of the citric acid cycle, including citric acid, succinic acid, fumaric acid, malic acid, and α-ketoglutarate, showed a decrease or even a significant down-regulation in abundance from the green fruit stage to the fully ripe stage. Notably, α-ketoglutarate, an endogenous intermediate metabolite, plays a pivotal role in organic acid and carbohydrate metabolism [40]. The reduction in these organic acids suggests that the citric acid cycle ultimately influences the taste and acidity of litchi fruit during its maturation process.

Amino acid biosynthesis stands as the foremost pathway in amino acid metabolism [24]. Throughout the development of XJF litchi, a total of 24 DAMs were involved in amino acid biosynthesis, with 19 up-regulated compounds encompassing most amino acids. The accumulation of phenylalanine, proline, leucine, and isoleucine not only imbued the fruit with a sweet and sour taste and abundant nutrition but was also closely correlated with the enhancement of the fruit’s antioxidant activity [41]. Concurrently with the up-regulation of amino acid metabolites, there was a down-regulation of organic acids such as α-ketoglutarate, isocitric acid, 2-isopropyl malic acid, and shikimic acid. This may be attributed to these organic acid metabolites serving as the substrates for most amino acid production [42]. Alanine stands as one of the most prevalent amino acids in numerous fruits [43], including litchi, and plays an essential role in glucose and tryptophan metabolism [44]. In this study, L-alanine exhibited up-regulation across all developmental stages.

## 5. Conclusions

In this study, we employed metabolomics to analyze the metabolites of XJF litchi at various developmental stages, with a particular focus on the content and types of metabolites associated with taste that accumulate from the green fruit stage to the commercial maturity stage. Our findings revealed the presence of a total of 985 metabolites, among which 238 taste-related metabolites were involved in the metabolic process. Cluster analysis and PCA showed that the composition and content of metabolites changed significantly in different developmental stages, which was closely related to developmental stages. Although the total metabolite abundance was notably higher during the green fruit stage compared to the color transition and commercial maturity stages, the total taste metabolite abundance was significantly lower than that during the S2 and S3 stages. The total sugar content steadily increased, yet monosaccharides and disaccharides exhibited distinct behaviors, highlighting the characteristic accumulation of reducing sugars. As the fruit developed, particularly from the green fruit stage to the color transition stage, most organic acids demonstrated a notable downward trend, with the abundance of total organic acids dropping to extremely low levels during the color transition and maturity stages. Conversely, the abundances of total amino acids, basic amino acids, and essential amino acids exhibited an upward trend. Eight essential amino acids and five flavor amino acids were detected, with most showing significantly higher abundances at the color transition and maturity stages. The number of DAMs across the three developmental stages exhibited a trend of S1 vs. S3 > S1 vs. S2 > S2 vs. S3. Through KEGG functional annotation and enrichment analysis, it was revealed that numerous taste-related metabolic pathways are present during the development of XJF litchi. Among these, amino acid biosynthesis, D-amino acid metabolism, 2-oxocarboxylic acid metabolism, glyoxylic acid and dicarboxylic acid metabolism, pentose phosphate pathway, tricarboxylic acid cycle, and carbon metabolism stand out as the most significantly enriched pathways, second only to secondary metabolite biosynthesis. Additionally, numerous metabolic pathways and taste DAMs observed from the green fruit stage to the color transition stage indicate that this critical phase lays a solid foundation for litchi taste development. This study contributes to a deeper understanding of the molecular mechanisms that govern taste and nutrient accumulation and metabolism during the development and maturation of litchi.

## Data Availability

The original contributions presented in the study are included in the article/Supplementary Material, further inquiries can be directed to the corresponding author.

## Supplementary Materials

The following supporting information can be downloaded at https://www.mdpi.com/article/10.3390/foods14010144/s1: Figure S1: Verification diagrams of OPLS-DA for comparison groups at different developmental stages. Figure S2: OPLS-DA score plots for comparison groups at different developmental stages. Figure S3: PCA plots for comparison groups at different developmental stages. Table S1: Identification information of 238 taste metabolites in the pulp of ‘XianJinFeng’ litchi. Table S2: DAMs of taste metabolites in group S1 vs. S2. Table S3: DAMs of taste metabolites in group S2 vs. S3. Table S4: DAMs of taste metabolites in group S1 vs. S3. Table S5: Common DAMs of taste metabolites in 3 comparison groups. Table S6: Information on KEGG metabolic pathways related to taste metabolism in XJF litchi during different developmental stages.
