# Supplementary material for: Metabolomics Unveiled the Accumulation Characteristics of Taste Compounds During the Development and Maturation of Litchi Fruit
Source: Foods. 2025 Jan 6;14(1):144. doi: 10.3390/foods14010144 (PMC11720449; doi:10.3390/foods14010144)
Supplement: Supplementary file 1 [file foods-14-00144-s001.zip › 2-Supplementary figure.pdf]

Supplementary figure

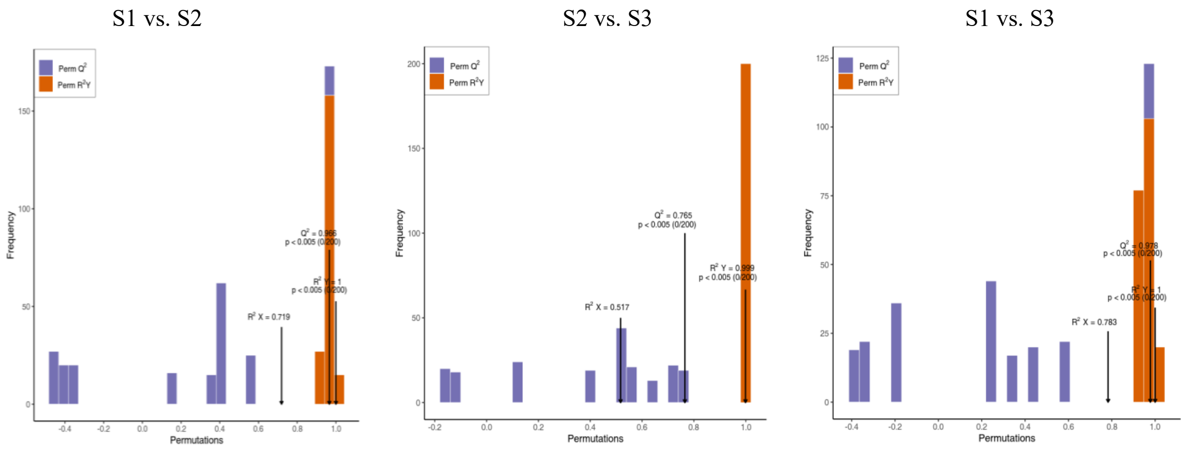

Figure S1 Verification diagrams of OPLS-DA for grouping comparison at different developmental stages

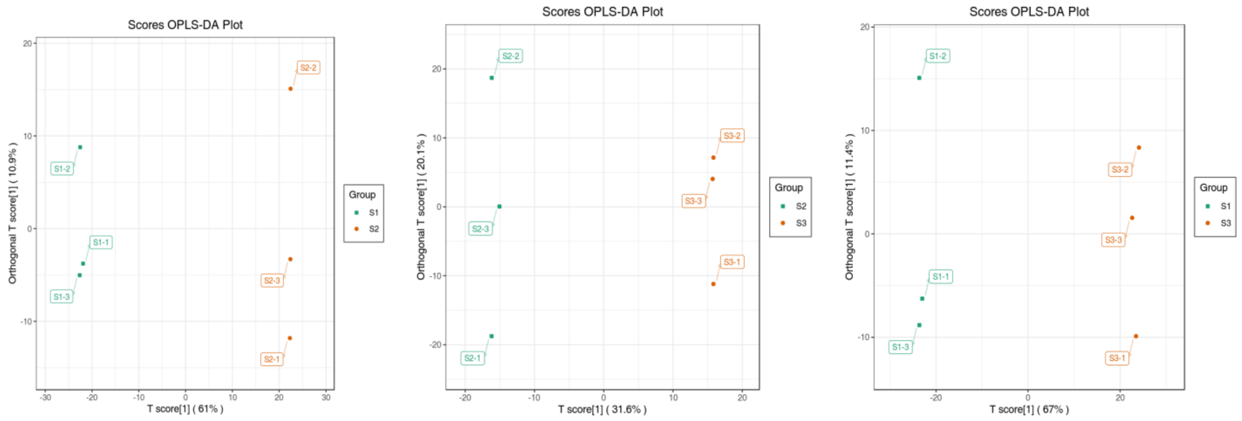

Figure S2 OPLS-DA score plots for grouping comparison at different developmental stages

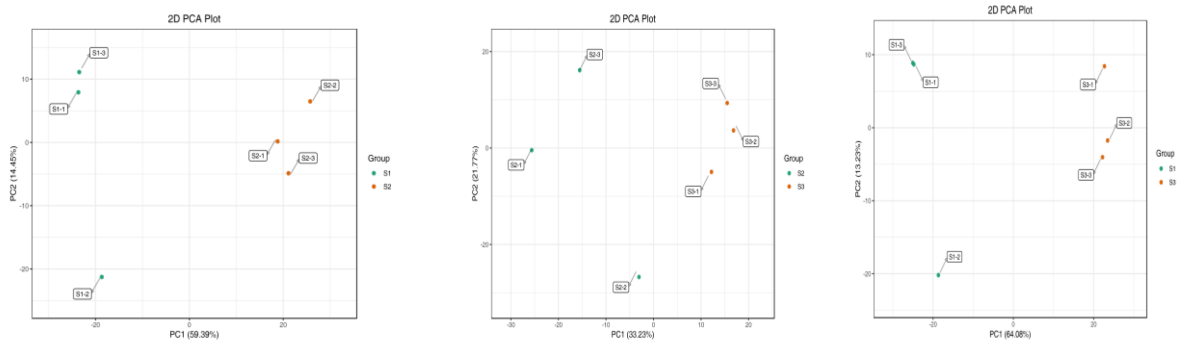

Figure S3 PCA plots for grouping comparison at different developmental stages
